# Prognostic factors and relapse in nodal vs. extranodal non-Hodgkin lymphoma of the ENT region: a prospective cohort study

**DOI:** 10.25122/jml-2025-0064

**Published:** 2025-04

**Authors:** Raluca Morar, Norberth-Istvan Varga, Claudia Raluca Balasa Virzob, Nicolae Constantin Balica, Ioana Delia Horhat, Alexandru Chioreanu, Oana Silvana Sarau, Sonia Tanasescu, Razvan Susan, Ion Cristian Mot

**Affiliations:** 1 Department of General Medicine, Doctoral School, Victor Babes University of Medicine and Pharmacy, Timisoara, Romania; 2 Department of Clinic Nursing, Victor Babes University of Medicine and Pharmacy, Timisoara, Romania; 3 ENT Department, Victor Babes University of Medicine and Pharmacy, Timisoara, Romania; 4 Department of Otorhinolaryngology, Vasile Goldis Western University of Arad, Arad, Romania; 5 Hematology Department, Municipal Emergency Clinical Hospital, Timisoara, Romania; 6 Department of Pediatrics, Victor Babes University of Medicine and Pharmacy, Timisoara, Romania; 7 Department of Family Medicine, Centre for Preventive Medicine, Victor Babes University of Medicine and Pharmacy, Timisoara, Romania

**Keywords:** Non-Hodgkin Lymphoma, lymph node, extranodal lymphoma, ENT, Ears, Nose and Throat, ENT lymphoma, NHL relapses, NHL prognostic, ENT, Ears, Nose and Throat, NHL, Non-Hodgkin Lymphoma, DLBCL, Diffuse Large B-cell Lymphoma, HR, Hazard Ratio

## Abstract

Non-Hodgkin Lymphoma (NHL) arising from the ear, nose, and throat (ENT) region presents unique challenges with regard to diagnosis and treatment. This study investigated the clinical characteristics, prognostic factors, and relapse patterns in patients with NHL originating from lymph nodes (nodal NHL) or other extranodal structures, aiming to identify factors associated with relapse between these two groups. This prospective cohort study included 50 patients diagnosed with NHL in the ENT region at a tertiary hospital in South-Western Romania between 2019 and 2021. Patients were categorized as having nodal or extranodal disease based on histopathological examination and were followed for three years to assess disease evolution, including relapse. Cox proportional hazards regression analysis was employed to identify factors associated with relapse-free survival. Extranodal NHL was associated with a significantly higher prevalence of multiple-site involvement compared to nodal NHL (53.3% vs. 30%, *P* = 0.021). While a trend towards increased relapse was observed in extranodal NHL, this was not statistically significant (*P* = 0.125). The presence of disseminated disease (HR = 27.295; *P* < 0.001) and undergoing only a biopsy (compared to total excision, HR = 4.301; *P* = 0.027) were identified as independent predictors of relapse. Kaplan-Meier analysis demonstrated significantly different relapse-free survival patterns among groups stratified by NHL localization and dissemination status (*P* < 0.001). The extent of surgical intervention is a crucial factor influencing relapse risk in ENT NHL, with total excision associated with a lower hazard of relapse. At the same time, extranodal involvement may indicate a more aggressive disease course, particularly when combined with dissemination. However, larger studies with longer follow-ups are needed to validate these findings and refine treatment strategies, especially in regions with limited access to healthcare and screening programs.

## INTRODUCTION

Lymphomas, a diverse group of malignancies from the lymphatic system, represent approximately 4–5% of all cancers diagnosed globally [1-3]. These cancers originate in lymphocytes, specialized white blood cells crucial for immune function, and can manifest in lymph nodes, the spleen, bone marrow, and other organs [1-4]. Due to their potential for widespread dissemination and impact on vital functions, lymphomas represent a significant global health concern, necessitating a comprehensive understanding of their underlying biology and clinical behavior. The two major classifications of lymphomas are Hodgkin lymphoma (HL) and non-Hodgkin lymphoma (NHL) [4,5]. While the presence of Reed-Sternberg cells is the hallmark of Hodgkin lymphoma, their absence defines the more prevalent and diverse group of malignancies known as non-Hodgkin lymphoma [5,6]. NHL encompasses a broad spectrum of cancers, each with unique characteristics arising from their distinct cellular origins within the B-cell, T-cell, or, rarely, natural killer (NK) cell lineages [6,7]. This heterogeneity manifests in a wide range of clinical presentations, varying degrees of aggressiveness, and differing responses to treatment [8]. Accurate subtyping of NHL, therefore, is of paramount importance for guiding prognosis and selecting appropriate therapeutic strategies [9].

Further complicating the landscape of NHL is the occurrence of extranodal disease, where lymphoma arises in organs outside the traditional lymphatic system. Nodal NHL, primarily within lymph nodes, often manifests as a localized or regional disease [10]. In the head and neck, the cervical lymph nodes are common sites for nodal NHL, serving as the initial point of detection in a significant proportion of cases [11]. These nodes, strategically positioned along the lymphatic drainage pathways of the head and neck, can be affected by both primary nodal NHL and the spread of lymphoma from extranodal sites [12]. The involvement of cervical lymph nodes in NHL can present with a palpable mass, often accompanied by symptoms such as pain, tenderness, or discomfort [10-14]. The extent of nodal involvement, as assessed through clinical examination and imaging studies, plays a crucial role in staging and determining the appropriate treatment approach [15]. Extranodal NHL, on the other hand, arises in non-lymphatic tissues, posing unique diagnostic and therapeutic challenges [16]. In the head and neck region, extranodal NHL can involve various sites, including the upper respiratory tract, such as the nasal cavity, paranasal sinuses, nasopharynx, and larynx; Waldeyer’s ring, comprising the palatine tonsil, lingual tonsil, and oropharyngeal wall; the salivary glands, including the parotid, submandibular, sublingual, and minor salivary glands; and the thyroid gland, typically with minor or no involvement of lymph nodes [17-19]. The diverse anatomical locations and functional implications of extranodal NHL in the head and neck underscore the need to thoroughly understand its specific characteristics and prognostic factors.

The diagnosis of NHL in the ENT region is often complicated by nonspecific symptoms, such as nasal obstruction, hearing loss, or sore throat, which can mimic various benign conditions, potentially delaying diagnosis [19]. Furthermore, the complex anatomy of the ENT region, with its proximity to critical structures like cranial nerves, major blood vessels, and the airway, poses significant challenges for treatment planning and can impact surgical approaches, radiation fields, and, ultimately, patient outcomes [19-21]. The potential for NHL involvement to affect vital functions such as speech, swallowing, and breathing further underscores the clinical significance of this disease entity.

Despite the importance of NHL in this anatomical location, there remains a paucity of research specifically addressing the nuances of nodal versus extranodal disease within the ENT region. Specifically, a deeper understanding of prognostic factors that predict disease progression and treatment response, including the impact of disease localization, histological subtype, and the role of surgical intervention, is needed. This study investigated the relationship between various factors and the risk of relapse in nodal and extranodal NHL located in the ENT region. The primary objective was to compare nodal and extranodal NHL groups regarding patient demographics, disease characteristics, and treatment modalities. The secondary objective was to identify significant prognostic relapse-related factors, including metastases, localization, and treatment approach.

## MATERIAL AND METHODS

### Study design and population

This observational, prospective cohort study was conducted at the ENT (Ears, Nose, and Throat) ward of the Timisoara Municipal Emergency Clinical Hospital. This hospital serves five counties in south-western Romania, with a population of more than two million people. Patient recruitment for this study occurred over two years, between October 1, 2019, and October 1, 2021. Each patient was followed for three years, with check-ups at 3, 6, 12, 24, and 36 months post-admission. The follow-up period ended on October 1, 2024.

Inclusion criteria were adult patients (age 18 years or older), confirmed diagnosis of NHL with cervical lymph node involvement or another ENT localization based on histopathological examination, complete data in the examination records, and provision of informed consent to participate in the study. Patients under 18 years of age, those who did not consent, and those with Hodgkin lymphoma were excluded. The recruited patients were categorized into two groups based on the site of the disease: those with NHL in cervical lymph nodes and those with NHL at other extranodal ENT sites.

### Data collection

Baseline demographic data, including age, sex, and place of residence (rural or urban), were recorded at the first hospital admission. Disease characteristics were assessed through histopathological examination of biopsy specimens and clinical staging. These included the primary site of NHL categorized as nodal or extranodal, with specific extranodal sites documented; histological subtype, classified according to the World Health Organization (WHO) classification system; and disease stage, determined using the Ann Arbor staging system. Extranodal localization was defined as the NHL that originated from the extranodal tissues of the head and neck region, including the nasal cavity, paranasal sinus, nasopharynx, larynx, palatine and lingual tonsils, oropharyngeal wall, thyroid gland, the parotid gland, and the submandibular, sublingual, and minor salivary glands. Nodal localization was defined as NHL originating in the lymph nodes. The presence of dissemination beyond the primary location was also noted. Treatment modalities recorded were the type of surgical intervention (biopsy only or total excision) and whether the patient received chemotherapy and/or radiotherapy. Disease evolution was monitored through scheduled check-ups for the next 36 months, which included physical examinations and imaging studies. The occurrence of relapse during the 3-year follow-up period was documented, with time to relapse calculated as the number of months from the end of the last chemo- and/or radiotherapy session to the date of relapse. The occurrence of death was also recorded. At the end of the follow-up period, each patient underwent a full-body CT scan.

### Statistical analysis

Descriptive statistics were used to summarize patient characteristics. The normality of age distribution was assessed using the Shapiro-Wilk test. To compare the ages of nodal and extranodal NHL groups, the Mann–Whitney U test was used due to the non-normal distribution of age. Associations between categorical variables were analyzed using the chi-square or Fisher's exact test, depending on the specific variables and sample size. To identify prognostic factors associated with relapse, a Cox proportional hazards regression analysis was performed. The time-to-event variable was defined as the number of months from the end of the last chemotherapy and/or radiotherapy session to the occurrence of relapse. Patients without relapse during the 3-year follow-up were censored at 36 months. Kaplan–Meier survival curves were generated to visually compare relapse-free survival between the nodal and extranodal NHL groups. *P* values below 0.05 were considered statistically significant. Data was analyzed using SPSS version 26.

## RESULTS

### General population characteristics

The study cohort consisted of 50 patients diagnosed with non-Hodgkin lymphoma in the ENT region. Two patients refused to consent to data collection and were excluded from this study. In terms of baseline characteristics, when the data collection process began, the sample comprised 24 men and 26 women, with 22 individuals residing in rural areas and 28 in urban areas. The mean age at the initial consultation was 60.38 years (SD = 14.419), ranging from 25 to 86 years. At the time of diagnosis, 20 patients presented with lymph node involvement, while 30 presented with extranodal NHL, with the primary site of origin being the palatine tonsil (19 patients), paranasal sinus (one patient), nasopharynx (seven patients), and tongue base (three patients). The most common histological subtypes were diffuse large B-cell lymphoma (DLBCL) (66%) and follicular lymphoma (22%), while T-cell lymphoma accounted for 12% of the cases.

Further data were collected during the follow-up period to assess disease characteristics, treatment modalities, and evolution. The distribution of patients across disease stages was as follows: II A (four patients), II B (five patients), III A (seven patients), III B (ten patients), IV A (ten patients), and IV B (14 patients). Chemotherapy was administered in all 50 cases, while radiotherapy was utilized in only two cases. Disease evolution was stationary (i.e., no relapse was recorded after treatment with chemotherapy) for 26 patients, while 23 patients had a relapse, and one death was recorded. Table 1 offers an overview of the study population.

**Table 1:** Study population characteristics

Age, mean (SD)	60.38 (14.41)
**Sex, *n* (%)** Male Female	24 (48) 26 (52)
**Provenience, *n* (%)** Urban Rural	28 (56) 22 (44)
**NHL localization, *n* (%)** Lymph-node Palatine tonsil Nasopharynx Paranasal sinus Tongue base	20 (40) 19 (38) 7 (14) 1 (2) 3 (6)
**Disease Stage, *n* (%)** II A II B III A III B IV A IV B	4 (8) 5 (10) 7 (14) 10 (20) 10 (20) 14 (28)
**Histological Subtype, *n* (%)** T-cell lymphoma DLBCL* Follicular lymphoma	6 (12) 33 (66) 11 (22)
**Surgical intervention, *n* (%)** Only biopsy Total excision	35 (70) 15 (30)
**Chemo-, Radio-therapy, *n* (%)** Chemotherapy Radiotherapy	50 (100) 2 (4)
**Evolution, *n* (%)** Stationary Relapse Death	26 (52) 23 (46) 1 (2)

* DLBCL, Diffuse Large B-cell Lymphoma

### Comparison of nodal and extranodal non-Hodgkin lymphoma

Table 2 compares nodal and extranodal NHL groups regarding patient demographics, disease characteristics, and treatment modalities.

Comparison between the nodal and extranodal NHL groups revealed no significant differences in age or sex distribution. Age, assessed using the Mann–Whitney U test due to non-normality (Shapiro–Wilk test, *P* = 0.033), did not differ significantly between the groups (*P* = 0.096). Similarly, sex was not significantly associated with NHL localization (Fisher's exact test, *P* = 0.159), indicating that men and women were equally likely to have nodal or extranodal NHL.

**Table 2:** Comparison of patient characteristics by NHL localization

Characteristic	Nodal NHL (*n* = 20)	Extranodal NHL (*n* = 30)	*P* value
**Age, mean (SD)**	55.65 (15.95)	63.53 (12.59)	0.096
**Sex, *n* (%)** Male Female	7 (35) 13 (35)	17 (56.7) 13 (43.3)	0.159
**Dissemination, *n* (%)**	6 (30)	16 (53.3)	0.021
**Disease Stage, *n* (%)** II A II B III A III B IV A IV B	2 (10) 2 (10) 3 (15) 5 (25) 5 (25) 3 (15)	2 (6.7) 3 (10) 3 (10) 6 (20) 5 (16.7) 11 (36.7)	0.128
**Histological Subtype, *n* (%)** T-cell NHL DLBCL Follicular NHL	3 (15) 10 (50) 7 (35)	3 (10) 23 (76.6) 4 (13.3)	0.129
**Radiotherapy, *n* (%)**	0 (0)	2 (6.7)	0.510
**Excision Surgery, *n* (%)**	3 (15)	12 (40)	0.069
**Relapse, *n* (%)**	6 (30)	17 (56.7)	0.086

Regarding disease characteristics, no significant association was found between NHL localization and histological type (chi-square test, χ^2^= 4.104, *P* = 0.129). Although this analysis did not reach statistical significance, it is important to note that the small number of patients in some stage groups may have limited our ability to detect an association. Descriptive analysis of the distribution of histological subtypes revealed that DLBCL was more common in extranodal NHL, while follicular lymphoma was more common in nodal NHL.

No significant association was found between NHL localization and disease stage (chi-square test, χ^2^= 7.157, *P* = .128). However, the small number of patients in the early stages of the disease limited the ability to draw definitive conclusions about the distribution of nodal and extranodal NHL across specific stages. Despite the lack of statistical significance, a potential trend was observed toward a higher proportion of extranodal NHL cases in disseminated lymphomas (stage IV B). No significant associations were found between NHL localization and the type of surgery performed (Fisher's exact test, *P* = 0.069), the administration of chemotherapy (Fisher's exact test, *P* = 0.400), or the administration of radiotherapy (Fisher's exact test, *P* = 0.510). Although a trend towards more total excision surgeries in the extranodal group was observed, this difference was not statistically significant. Similarly, no significant association was found between relapse and NHL localization (Fisher's exact test, *P* = 0.086), although a possible trend towards more relapses in the extranodal group was noted. The small number of patients receiving radiotherapy (2 out of 50) may have limited the power to detect a significant association with localization.

### Factors associated with relapse

A Cox proportional hazards regression analysis was performed to evaluate the effects of various factors on the time to relapse in NHL patients. The model, which included age, sex, localization, histological type, disease stage, surgery, and extranodal involvement as predictors, was statistically significant (likelihood ratio chi-square = 42.323, *P* < 0.001).

Several variables were identified as significant predictors of relapse. Dissemination was strongly associated with an increased hazard of relapse (HR = 27.295; *P* < 0.001). To assess the independent association of each histological subtype with relapse risk, separate Cox regression analyses were performed with each subtype as the sole predictor. None of the histological subtypes were significantly associated with relapse risk (all *P* values > 0.05). Specifically, the hazard ratio for T-cell lymphoma was 0.038 (*P* = 0.184), for DLBCL, it was 1.058 (*P* = 0.902), and for follicular lymphoma, it was 1.964 (*P* = 0.138). Disease stage was a significant predictor (*P* = 0.024), with stage IV B showing a higher hazard of relapse (HR = 1.059; *P* = 0.001) compared to stage II A. The type of surgical intervention was also a significant predictor of relapse, with total excision associated with a lower hazard of relapse compared to biopsy alone. Undergoing only a biopsy was associated with a higher hazard of relapse (HR = 4.301; *P* = 0.027). Neither age (HR = 1.002; *P* = 0.910) nor sex (HR = 0.785; *P* = 0.642) was significantly associated with relapse risk. The Cox regression analysis suggested that extranodal localization was associated with a higher hazard of relapse (HR = 3.068) compared to nodal localization, but this finding was not statistically significant (*P* = 0.125). Table 3 presents the results of the Cox regression analysis, showing the hazard ratios and *P* values for each predictor variable.

**Table 3:** Cox proportional hazards regression analysis of factors associated with relapse

Predictor variable	Hazard ratio (HR)	95% Confidence interval	*P* value
Age	1.002	(0.988, 1.016)	0.91
Sex	0.785	(0.461, 1.338)	0.642
Extranodal localization	3.068	(0.751, 9.521)	0.125
Histological Subtype T-cell NHL DLBCL Follicular NHL	0.038 1.058 1.964	(0.001, 1.412) (0.654, 1.713) (0.713, 5.412)	0.184 0.902 0.138
Dissemination	27.295	-	< 0.001
Biopsy only (vs. Total Excision)	4.301	-	0.027

**Figure 1:**
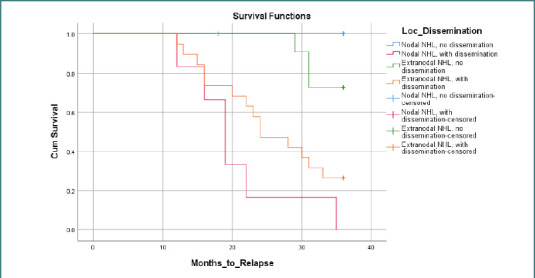
Relapse-free survival in nodal vs. extranodal NHL

To analyze the impact of both NHL localization and the presence of disseminated disease on relapse-free survival (RFS), a Kaplan–Meier survival analysis was conducted, stratifying the patient cohort into four distinct groups: (1) nodal NHL without dissemination; (2) nodal NHL with dissemination; (3) extranodal NHL without dissemination; (4) extranodal NHL with dissemination (Figure 1). The analysis revealed significant differences in RFS among these four groups (log-rank test, *P* < 0.001), indicating that the combination of localization and dissemination status influences RFS patterns. Specifically, extranodal NHL with dissemination exhibited the poorest RFS, while nodal NHL without dissemination demonstrated the best RFS. The remaining two groups, extranodal NHL without dissemination and nodal NHL with dissemination, showed intermediate RFS. These findings underscore the importance of considering both NHL localization and the presence of disseminated disease when assessing relapse risk.

## DISCUSSION

Our study investigated the characteristics and prognostic factors associated with relapse in a cohort of 50 patients with NHL in the ENT region. The primary objective was to compare nodal and extranodal NHL, while the secondary objective was to identify factors associated with relapse.

Our analysis revealed that patients with extranodal NHL were more likely to be involved in multiple sites than those with nodal NHL (53.3% vs. 30%, *P* = 0.021). This finding aligns with Shi *et al*., who observed a higher frequency of involvement of multiple sites in extranodal diffuse large B-cell lymphoma (DLBCL) compared to nodal DLBCL [22]. Given the higher prevalence of more widespread disease, our Cox regression analysis demonstrated that extranodal localization was associated with a higher hazard of relapse (HR = 3.068), although this finding did not reach statistical significance (*P* = 0.125). The *P* value indicates that this observed difference could be due to the small study population. Similarly, Mian *et al*. found inferior overall survival in extranodal DLBCL compared to nodal DLBCL [23]. This suggests that extranodal involvement might be a marker of a more aggressive disease or indicate a poorer response to treatment.

However, it is important to consider that our study, unlike previous research focusing specifically on DLBCL [23], included various histological subtypes of NHL. This heterogeneity may explain the discrepancy between our finding of a trend towards worse relapse-free survival in extranodal NHL and the similar benefits of R-CHOP reported by Olszewski *et al*. for both nodal and extranodal DLBCL [24]. Similarly, while Lee *et al*. found better disease-specific survival in extranodal DLBCL [25], our study assessed relapse due to a lack of mortality events, and survival and relapse are distinct concepts.

Our findings on the prevalence of extranodal lymphoma in the head and neck region are consistent with Bojanowska-Poźniak *et al*., who found that 73% of cases presented extranodal [26]. Their study further highlights the diverse range of extranodal sites involved. While our study found a trend towards worse relapse-free survival in extranodal NHL, Qi *et al*. reported similar prognoses for DLBCL arising in the Waldeyer's ring and lymph nodes [27]. This discrepancy may be attributed to the heterogeneity of our cohort.

Finally, Yan *et al*. conducted a similar study focusing on extranodal NHL in the head and neck region, identifying DLBCL as the most prevalent subtype [28]. However, their study reported a significantly lower proportion of extranodal cases (36.66%) compared to our findings (60%). This difference may reflect variations in patient populations or diagnostic criteria.

The Cox regression analysis identified the involvement of multiple sites as the strongest predictor of relapse (HR = 27.295; *P* < 0.001). Interestingly, the type of surgery performed was also significantly associated with relapse risk. Patients who underwent only biopsy had a higher hazard of relapse compared to those who underwent total excision surgery (HR = 4.301; *P* = 0.027). This suggests that more extensive surgical resection might contribute to a lower relapse risk, although further research is needed to confirm this observation. Our Kaplan–Meier survival analysis, which stratified the cohort by both NHL localization and the presence of disseminated disease, revealed significant differences in relapse-free survival (RFS) among the four resulting groups. Extranodal NHL with dissemination was associated with the poorest RFS, while nodal NHL without dissemination had the best RFS. This result underscores the complex interplay between the primary site of NHL origin and the extent of disease spread. It suggests that while extranodal NHL might not inherently carry a worse prognosis, the combination of extranodal involvement and disseminated disease appears to be a significant risk factor for relapse.

Our study has several strengths, including its prospective design, detailed data collection, and the use of Cox regression analysis to assess prognostic factors for relapse. However, it also has limitations. The small sample size might have limited the statistical power to detect significant associations for some variables. The small number of patients in some stage groups limited our ability to draw definitive conclusions about nodal and extranodal NHL distribution within those specific stages. Future studies with larger sample sizes should stratify analyses by stage to more thoroughly investigate the influence of disease stage on outcomes in nodal versus extranodal NHL, particularly in the ENT region. Furthermore, our follow-up period of just 3 years might not be sufficient for the complete evaluation of relapse, as some relapses might occur after this period. This limited follow-up also impacted our ability to assess survival outcomes, as only one death occurred in our cohort. This precluded any meaningful statistical analysis on survival and restricted our focus to relapse as the primary endpoint. A longer follow-up period might have yielded more deaths due to NHL and allowed for a more comprehensive analysis of both relapse and survival. However, it is important to note that this 3-year follow-up period was the timeframe for which ethical approval was granted by our hospital's ethics committee. Further research with larger sample sizes and more extended follow-up periods is needed to confirm our findings and explore the complex interplay of factors influencing relapse risk in nodal and extranodal NHL of the ENT region.

Another noteworthy limitation of our study is the high proportion of patients who presented with late-stage disease, particularly those from rural areas (22 out of 50 patients). This likely reflects the limited access to specialized medical care and the lack of effective screening programs for lymphoma in these regions within the five counties of South-Western Romania served by our hospital. The delayed diagnosis inherent in the late-stage presentation may have led to a selection bias, where our cohort is skewed towards more aggressive or symptomatic cases of NHL in the ENT region. Consequently, our findings may not be fully generalizable to all patients with ENT NHL, particularly those with early-stage disease or those residing in areas with better access to healthcare and screening.

## CONCLUSION

This study examined clinical characteristics and relapse patterns in patients with nodal and extranodal NHL of the ENT region. Our findings indicate that extranodal NHL in this region is associated with a higher prevalence of disseminated disease compared to nodal presentations. While a trend towards increased relapse in extranodal NHL was observed, this did not reach statistical significance, possibly due to the limited sample size. The extent of surgical resection was identified as a significant predictor of relapse, with total excision associated with a significantly lower hazard of relapse compared to biopsy alone. Furthermore, the presence of disseminated disease was identified as the strongest independent predictor of relapse. Cox regression analysis demonstrated that patients with disseminated disease had a significantly higher hazard of relapse compared to those without. Additionally, the Kaplan–Meier survival analysis revealed significant differences in relapse-free survival patterns among groups stratified by NHL localization and dissemination status. Specifically, the combination of extranodal involvement and disseminated disease was associated with the poorest relapse-free outcomes. These findings provide further evidence for the importance of considering disease localization, extent of surgical intervention, and dissemination status when assessing and managing NHL in this region. Future research, including larger studies with longer follow-up periods and more comprehensive analyses of the effects of various therapeutic strategies, is needed to validate these results and inform more effective management strategies for patients with nodal and extranodal NHL in the ENT region, especially those from underserved areas.

## Data Availability

Data are contained within this article.

## References

[1] Matasar MJ, Zelenetz AD (2008). Overview of lymphoma diagnosis and management. Radiol Clin North Am.

[2] Bispo JAB, Pinheiro PS, Kobetz EK (2020). Epidemiology and Etiology of Leukemia and Lymphoma. Cold Spring Harb Perspect Med.

[3] Roman E, Smith AG (2011). Epidemiology of lymphomas. Histopathology.

[4] Mugnaini EN, Ghosh N (2016). Lymphoma. Prim Care.

[5] Cheson BD, Fisher RI, Barrington SF, Cavalli F, Schwartz LH, Zucca E, Lister TA, Alliance, Australasian Leukaemia and Lymphoma Group; Eastern Cooperative Oncology Group; European Mantle Cell Lymphoma Consortium; Italian Lymphoma Foundation; European Organisation for Research; Treatment of Cancer/Dutch Hemato-Oncology Group; Grupo Español de Médula Ósea; German High-Grade Lymphoma Study Group; German Hodgkin's Study Group; Japanese Lymphorra Study Group; Lymphoma Study Association; NCIC Clinical Trials Group; Nordic Lymphoma Study Group; Southwest Oncology Group; United Kingdom National Cancer Research Institute (2014). Recommendations for initial evaluation, staging, and response assessment of Hodgkin and non-Hodgkin lymphoma: the Lugano classification. J Clin Oncol.

[6] Lewis WD, Lilly S, Jones KL (2020). Lymphoma: Diagnosis and Treatment. Am Fam Physician.

[7] Sapkota S, Shaikh H (2023). Non-Hodgkin Lymphoma. StatPearls [Internet].

[8] Singh R, Shaik S, Negi BS, Rajguru JP, Patil PB, Parihar AS, Sharma U (2020). Non-Hodgkin's lymphoma: A review. J Family Med Prim Care.

[9] Ansell SM (2015). Non-Hodgkin Lymphoma: Diagnosis and Treatment. Mayo Clin Proc.

[10] Armitage JO, Weisenburger DD (1998). New approach to classifying non-Hodgkin's lymphomas: clinical features of the major histologic subtypes. Non-Hodgkin's Lymphoma Classification Project. J Clin Oncol.

[11] Weber AL, Rahemtullah A, Ferry JA (2003). Hodgkin and non-Hodgkin lymphoma of the head and neck: clinical, pathologic, and imaging evaluation. Neuroimaging Clin N Am.

[12] Luo J, Craver A, Bahl K, Stepniak L, Moore K, King J (2022). Etiology of non-Hodgkin lymphoma: A review from epidemiologic studies. J Natl Cancer Cent.

[13] Chiu BC, Hou N (2015). Epidemiology and etiology of non-hodgkin lymphoma. Cancer Treat Res.

[14] Bisig B, Savage KJ, De Leval L (2023). Pathobiology of nodal peripheral T-cell lymphomas: current understanding and future directions. Haematologica.

[15] Cheson BD, Pfistner B, Juweid ME, Gascoyne RD, Specht L, Horning SJ (2007). International Har-monization Project on Lymphoma. Revised response criteria for malignant lymphoma. J Clin Oncol.

[16] Freeman C, Berg JW, Cutler SJ (1972). Occurrence and prognosis of extranodal lymphomas. Cancer.

[17] Yang H, Xun Y, Ke C, Tateishi K, You H (2023). Extranodal lymphoma: pathogenesis, diagnosis and treatment. Mol Biomed.

[18] Paes FM, Kalkanis DG, Sideras PA, Serafini AN (2010). FDG PET/CT of extranodal involvement in non-Hodgkin lymphoma and Hodgkin disease. Radiographics.

[19] Weber AL, Rahemtullah A, Ferry JA (2003). Hodgkin and non-Hodgkin lymphoma of the head and neck: clinical, pathologic, and imaging evaluation. Neuroimaging Clin N Am.

[20] Etemad-Moghadam S, Tirgary F, Keshavarz S, Alaeddini M (2010). Head and neck non-Hodgkin's lymphoma: a 20-year demographic study of 381 cases. Int J Oral Maxillofac Surg.

[21] Urquhart A, Berg R (2001). Hodgkin's and non-Hodgkin's lymphoma of the head and neck. Laryngoscope.

[22] Shi Y, Han Y, Yang J, Liu P, He X, Zhang C (2019). Clinical features and outcomes of diffuse large B-cell lymphoma based on nodal or extranodal primary sites of origin: Analysis of 1,085 WHO classified cases in a single institution in China. Chin J Cancer Res.

[23] Mian M, Capello D, Ventre MB, Grazio D, Svaldi M, Rossi A (2014). International Extranodal Lymphoma Study Group (IELSG). Early-stage diffuse large B cell lymphoma of the head and neck: clinico-biological characterization and 18 year follow-up of 488 patients (IELSG 23 study). Ann Hematol.

[24] Olszewski AJ, Winer ES, Castillo JJ (2014). Improved survival with rituximab-based chemoimmunotherapy in older patients with extranodal diffuse large B-cell lymphoma. Leuk Res.

[25] Lee DY, Kang K, Jung H, Park YM, Cho JG, Baek SK (2019). Extranodal involvement of diffuse large B-cell lymphoma in the head and neck: An indicator of good prognosis. Auris Nasus Larynx.

[26] Bojanowska-Poźniak K, Nurkowska M, Danilewicz M, Pietruszewska W (2017). Clinical manifestation of malignant lymphomas of the head and neck region. Otolaryngol Pol.

[27] Qi SN, Li YX, Wang H, Wang WH, Jin J, Song YW (2009). Diffuse large B-cell lymphoma: clinical characterization and prognosis of Waldeyer ring versus lymph node presentation. Cancer.

[28] Yan S, Ma J, Yang M, Liu B, Li S, Yang L (2022). Analysis of the Clinicopathologic Characteristics and Prognosis of Head and Neck Lymphoma. Anal Cell Pathol (Amst).

